# Flood Hazard Assessment of the Urban Area of Tabuk City, Kingdom of Saudi Arabia by Integrating Spatial-Based Hydrologic and Hydrodynamic Modeling

**DOI:** 10.3390/s19051024

**Published:** 2019-02-28

**Authors:** Ashraf Abdelkarim, Ahmed F. D. Gaber, Ahmed M. Youssef, Biswajeet Pradhan

**Affiliations:** 1 Research Center, Ministry of Housing, Riyadh 68222, Saudi Arabia; dr.ashrafgis2020@gmail.com; 2 Department of Geography, Faculty of Art, Sohag University, Sohag 82524, Egypt; adahy70@gmail.com; 3 Geography and GIS Department, College of Arts, Imam Abdulrahman Bin Faisal University, Dammam 31441, Saudi Arabia; 4 Department of Geology, Faculty of Science, Sohag University, Sohag 82524, Egypt; 5 Geological Hazards Department, Applied Geology Sector, Saudi Geological Survey, P.O. Box 54141, Jeddah 21514, Saudi Arabia; 6 Centre for Advanced Modelling and Geospatial Information Systems (CAMGIS), Faculty of Engineering and IT, University of Technology, Sydney, NSW 2007, Australia; Biswajeet.Pradhan@uts.edu.au; 7 Department of Energy and Mineral Resources Engineering, Choongmu-gwan, Sejong University, 209 Neungdong-ro, Gwangjin-gu, Seoul 05006, Korea

**Keywords:** sustainable urban development, remote sensing, GIS, hydrologic modeling, hydraulic modeling (HEC-RAS), flood hazard, Tabuk City, Kingdom of Saudi Arabia

## Abstract

This study deals with the use of remote sensing (RS), geographic information systems (GISs), hydrologic modeling (water modeling system, WMS), and hydraulic modeling (Hydrologic Engineering Center River Analysis System, HEC-RAS) to evaluate the impact of flash flood hazards on the sustainable urban development of Tabuk City, Kingdom of Saudi Arabia (KSA). Determining the impact of flood hazards on the urban area and developing alternatives for protection and prevention measures were the main aims of this work. Tabuk City is exposed to frequent flash flooding due to its location along the outlets of five major wadis. These wadis frequently carry flash floods, seriously impacting the urban areas of the city. WMS and HEC-HMS models and RS data were used to determine the paths and morphological characteristics of the wadis, the hydrographic flow of different drainage basins, flow rates and volumes, and the expansion of agricultural and urban areas from 1998 to 2018. Finally, hydraulic modeling of the HEC-RAS program was applied to delineate the urban areas that could be inundated with floodwater. Ultimately, the most suitable remedial measures are proposed to protect the future sustainable urban development of Tabuk City from flood hazards. This approach is rarely used in the KSA. We propose a novel method that could help decision-makers and planners in determining inundated flood zones before planning future urban and agricultural development in the KSA.

## 1. Introduction

Flash floods are among the most frequent events that cause substantial problems all over the world [1,2,3,4,5,6,7,8,9,10]. The amounts and rates of floodwater flow are related to interactions among many variables, including the frequency of rainfall and its properties; the morphometric properties of drainage basins (catchment shape, number and length of streams); the physical properties of soil (depth, texture, and hydraulic conductivity); land use and land cover characteristics; and anticipated conditions (soil moisture values) [11,12]. It is commonly known that unplanned urbanization increases the danger of flash flooding because it increases the extent of impermeable areas [13,14,15]. Flash floods are generated after high-intensity rainfall events, particularly on steep mountainous terrain and hilly slopes that are barren and lack vegetation [16], such as arid areas.

In the literature, various researchers have applied remote sensing (RS) and geographic information system (GIS) techniques to estimate flood hazard impacts on urban areas, infrastructures, and agricultural areas and with regard to land use changes [17,18,19,20,21,22,23]. Others have studied flood hazard susceptibility mapping using RS data and GIS techniques with the help of statistical, probabilistic, hydrologic, and stochastic neural networks and fuzzy logic [11,24,25,26,27,28,29,30,31,32,33,34,35]. In a recent paper, Essel [28] and Portugués-Molla et al. [36] indicated that hydrologic assessment using geospatial techniques could be conducted to identify different hydrologic components, prepare hydrologic designs, and develop possible scenarios to overcome the hazards from flash flooding.

In recent years, many areas around the globe have been experiencing unprecedented rainstorm events (above-normal records) due to climate change [2,37,38,39]. These events cause disasters that result in human, property, and economic loss. Unprecedented events have affected the Kingdom of Saudi Arabia (KSA), causing considerable damage to highways, railroads, urban zones, and agricultural areas [11]. Most of the flash flood hazards in the KSA are caused by a combination of natural conditions (heavy rainfall and climate change) and human interference (poor drainage systems and unplanned urban expansion). Recently, heavy rainfall events triggered flash floods in areas of the KSA (Jeddah City in 2009, 2011, 2015, 2017, and 2018 and Al Riyadh in 2015 and 2018). The severely hit areas were generally in the western part of the country, particularly in the city of Jeddah during November 2009 and January 2011 [11,12]. These events were characterized by 70 and 111 mm of rainfall within 3 h, respectively, and were considered catastrophic flash floods for Jeddah City. They caused a death toll of 113 people in 2009, and together they damaged more than 10,000 homes and destroyed approximately 17,000 vehicles. The Kingdom of Saudi Arabia faces various challenges in implementing sustainable development plans due to the limitation of water resources and inadequate flash flood mitigation measures. Several authors have addressed the flash flood problems in different parts of the KSA. El Shinnawy et al. [40] addressed flash floods in the design of sustainable development in the Alsail Alkabir area. Elkhrachv [41] mapped flash flood areas in Najran City using satellite images and GIS. Sharif et al. [42] studied flood hazards in urban watersheds in Riyadh City. Abdul Karim [43] identified the impact of flood hazards on urban growth and land use changes in Ha’il City. Al-Ghamdi et al. [13] studied the impact of urban expansion on flood hazard in Makkah City.

Tabuk is one of the Saudi cities that have pursued urban expansion that encroaches on the wadis, which means these portions of the city are located in areas vulnerable to flash floods. Tabuk City has expanded toward wadi areas, but detailed hydrologic studies are absent. Many flood projects that have been applied to local areas inside Tabuk City have not taken into account the dimensions of water pathways and streams. Accordingly, unexpected severe flood events in the city in 1981, 1988, 2008, 2010, 2012, 2013, and 2016 caused loss of life and property. Abushandi [44] and Embaby et al. [45] indicated that Tabuk City suffered severe flash floods in 1981, 1988, 2010, 2012, and January 2013, which caused damage to infrastructure and loss of life. This study was undertaken to evaluate the effective impact of flash floods on urban areas of Tabuk City, using an innovative approach for the KSA based on RS, GIS, the water modeling system (WMS), the Hydrologic Engineering Center Hydrologic Modeling System (HEC-HMS), and the Hydrologic Engineering Center River Analysis System (HEC-RAS). Alternative protective measures are proposed in the study area to help mitigate the impact of floods on Tabuk City.

## 2. Study Area

**Figure 1:**
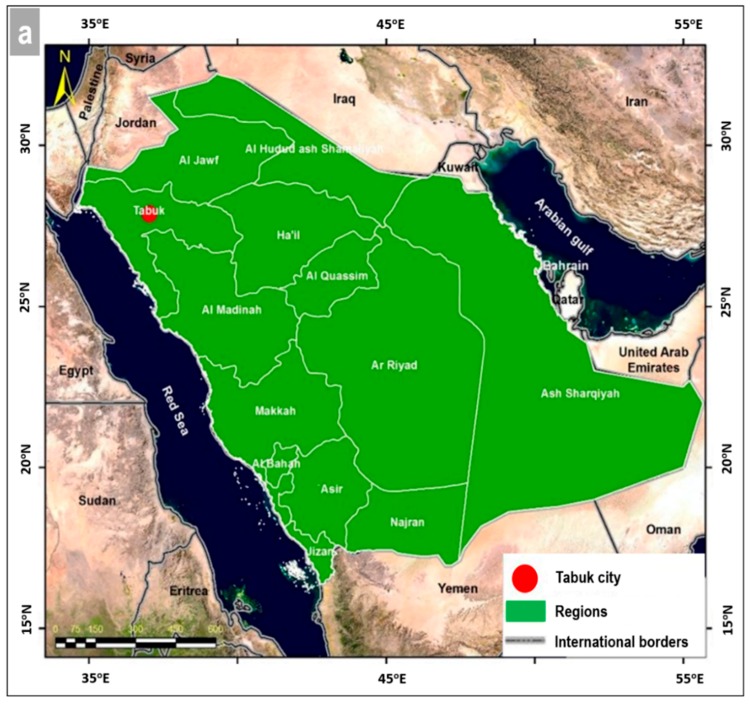

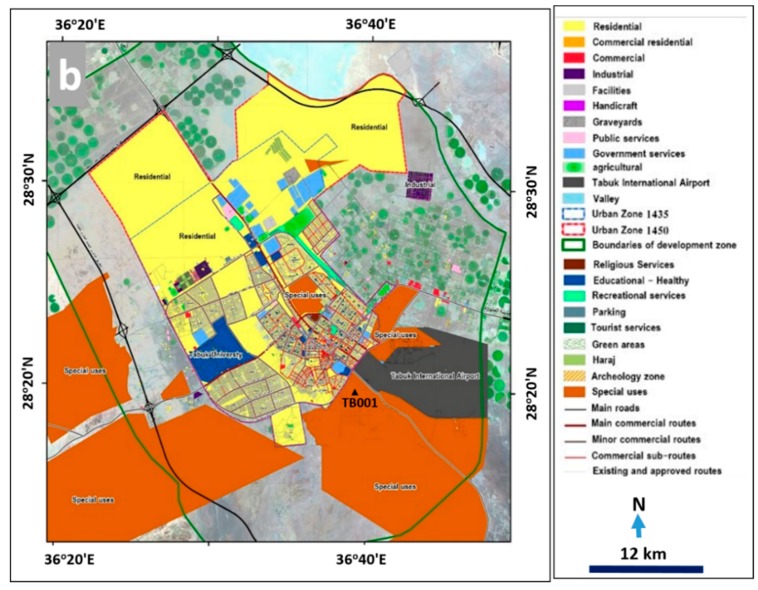
(**a**) Location of Tabuk City in the Kingdom of Saudi Arabia (KSA); (**b**) neighborhoods of Tabuk City, showing its planned urban expansion until 2030. (Source: Ministry of Municipal and Rural Affairs, Cities Planning Agency, General Administration for Urban Planning, Atlas of City Expansions, Tabuk City, No. 21356.).

Tabuk City is situated in the northwest of the KSA at latitude 28° 23′ 0.0″ N and longitude 36° 22′ 0.0″ E (Figure 1a), approximately 700 km northwest of Al-Madinah City. It represents the capital of the Tabuk region and the northern corridor of the KSA, which is one of the main gateways to surrounding countries and one of the KSA’s important national centers. Tabuk City also plays an important role as a fundamental location of the trend toward urban formation and growth. The city is growing to cover the low-lying plain between 800 and 1000 m above sea level, and many wadis run through the city. Geologically, the study area lies within the sedimentary cover of the Arabian shield [46,47]. The geology of the area comprises two categories: (1) the surface geology, which covers most of the study area and includes Quaternary sediments (silt and blown sand with pebbles of limestone and Pre-Cambrian rocks); and (2) the subsurface geology, which is characterized by two formations: (a) the Qasim Formation, with a thickness of about 500 m, which consists of sandstone, shale, and clay units of marine and continental origin, and (b) the Saq Formation, which covers a very large area with variable thickness, up to about 600 m thick in some places of the study area. The Saq Formation is composed of medium- to coarse-grained sandstone. The Qasim and Saq Formations appear as outcrops (from 100 to 150 m above ground level) along the southern and southwestern portions of the study area. 

The city has an arid climate with an annual maximum temperature of 28.7 °C, a minimum of 15.5 °C, and an average of 21.9 °C, with relative humidity of 33% on average. Average annual rainfall was calculated to be 39 mm, according the TB001 rain station (Figure 1b), and intense rainstorms occur, sometimes causing flash floods. The urban structure has a combined traditional texture, including main neighborhoods such as Khalediah, Al Saadah, Al Montazah, and Al Manshiyah (Figure 1b). There are modern urban blocks to the east and north, with a net texture and modern buildings in main neighborhoods such as Sultana, Al Sulaimaniya, Al Warood, and Al Mahrajan. These neighborhoods are located in the eastern, northern, and western outskirts of the city, while the slums are concentrated in the central and western parts of the city (Figure 1b). The cultivated lands surrounding Tabuk City are limited to the plains.

## 3. Causes and Consequences of Flash Floods in Tabuk City

**Figure 2:**
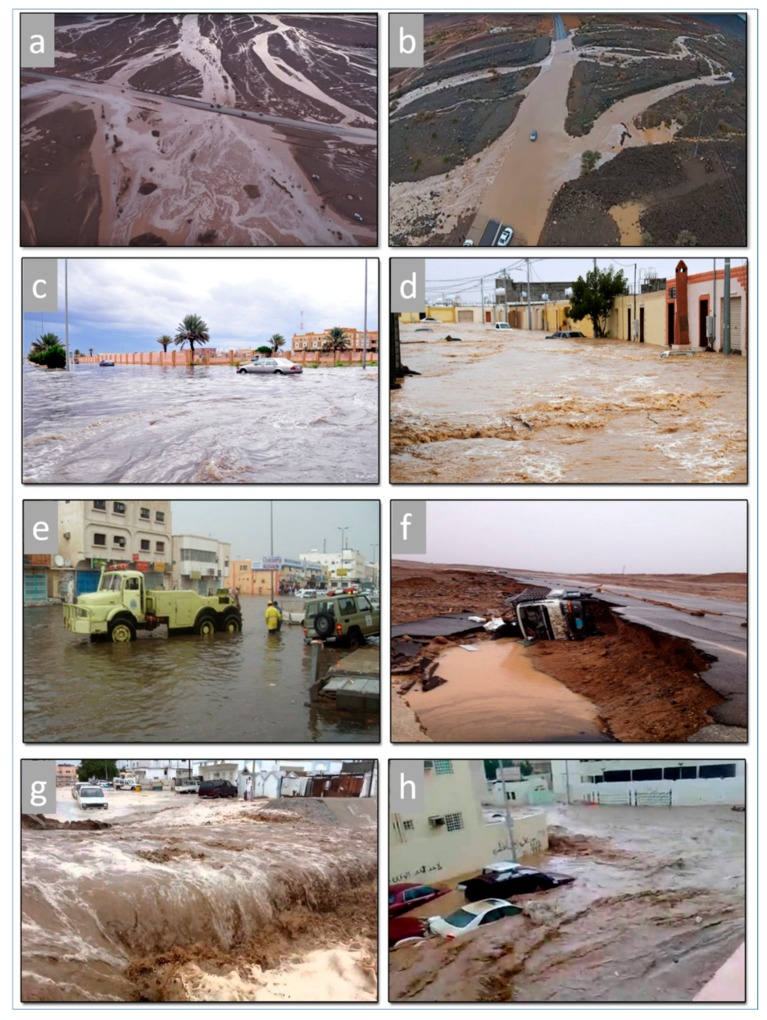
Field photos showing large portions of Tabuk City completely inundated with water and mud during flood events of 2016 (**a**–**d**) and 2013 (**e**–**h**). (**a**,**b**) Aerial photographs showing flooded areas; (**c**,**d**) flood water inundating the southern district of Tabuk City; (**e**–**h**) flash flood waters attacking the eastern district at a depth of more than 50 cm (note undercutting of the highway due to floodwaters in (**f**)).

Diachronic changes of urban areas and infrastructures can be seen in Tabuk City, which has expanded substantially in the last few decades. These urban and infrastructure expansions have reached flash flood–prone areas. The most important factor that has a significant impact on creating the danger of flash flooding is the improper use of land for urbanization where the paths of the wadis have been closed. Flash floodwaters in Tabuk City come from drainage basins and hills located south and southeast of the city. Runoff water follows the original paths toward the northwest, moving through the city. Due to unprecedented rainfall events, the frequency of flash floods has increased, causing an inundation hazard for the city. The severe flash floods of 1981, 1988, 2010, 2012, and 2013 were documented by many authors [44,45]. In addition, the city was hit by large floods in 2008 and 2016. The heavy rains of 2008, 2013, and 2016 paralyzed the city. The extreme inundation resulted in loss of life, damage to urban areas and infrastructures (main highways and roads), damage to hundreds of vehicles, and erosional effects along roads and wadi sediments, and it took people many hours to reach their homes. Figure 2 shows the impacts of flash floods that occurred in 2013 and 2016. According to the meteorological data, about 50 rainstorms occurred during each of those storms, lasting a few hours. Field and data analysis indicated that a number of factors contributed significantly to these flash flood events, including: (1) unprecedented severe rainfall; (2) inadequate wadi paths due to anthropogenic interference (urban expansion and encroachment across the paths); (3) insufficient drainage systems used to drain the floodwater (a design problem); (4) factors related to manmade infrastructure such as roads, highways, agricultural areas, and earth dikes that obstructed the path of the water; and (5) other factors related to the absence of detailed hydrologic studies, early warning systems, and engineering solutions for the upper reaches of the wadis.

## 4. Data and Methodology Used

Identifying the urban areas most exposed to flood hazards can be accomplished by using a variety of methods and assessment criteria. This process is complicated and requires a wide range of environmental, natural, meteorological, hydrologic, and hydraulic data in addition to quantitative and qualitative criteria in order to make a final decision. The integration of RS and GIS with WMS, HEC-HMS, and HEC-RAS is considered to be a suitable approach to support decision-making and planning procedures. These techniques were used together to guide sustainable development of Tabuk City, especially to reduce and avoid flash floods. 

**Figure 3:**
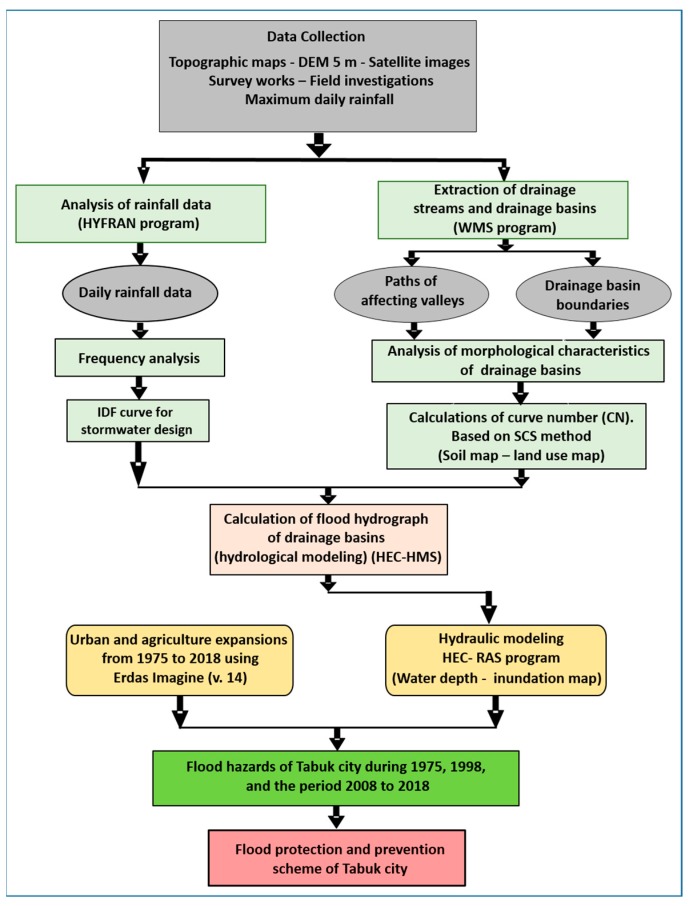
Flowchart showing the methodology adopted in the current work.

This study demonstrates the application of hydrologic and hydraulic models with the help of GIS and RS to determine the areas of Tabuk City most prone to flash flooding and the impact of flash floods on urban areas in the city (Figure 3). To achieve this, different datasets were used: (1) Topographic maps (1:50,000 scale). (2) Geologic maps (1:250,000 scale), used to understand different geologic units and soil hydrologic property types in the study area using ArcGIS. (3) A digital elevation model (DEM) at 5 m resolution, used for flood modeling. (4) Landsat images of different spatial resolutions, with Thematic Mapper (TM, 30 m), enhanced Thematic Mapper Plus (ETM+, 15 m), and Operational Land Imager (OLI, 15 m) used to extract images of agricultural and urban expansion, with the results verified using Geo-Eye images (1.84 m multispectral resolution). A supervised classification method (maximum likelihood classification) was used to extract land use maps from Landsat OLI images of the study area. In addition, remote sensing techniques were employed to monitor changes in agricultural and urban expansion from 1975 to 2018, and urban and agricultural sprawl in the study area for that time period was mapped. Urban and agricultural areas are typically mapped from digital remotely sensed data [48,49,50,51]. Lillesand and Kiefer [52] indicated that the main objective of the image classification procedure is to automatically categorize all pixels in images that relate to urban areas. (5) Rainfall records for the available stations. (6) Information on previous flood events from national databases, bibliographies, and field data, including photographs of inundated areas. The obtained datasets have different formats and scales, so to facilitate the precise processing and interaction between them, a GIS database using ArcGIS 10.2 software was developed. All datasets were georeferenced using the UTM coordinate system, zone 37. Digital elevation models and topographic maps were used to extract all streams and catchment areas from the images. Interpretation of satellite images was carried out to determine the areas of urban expansion, and HEC-RAS was used to determine the inundation zones. Finally, GIS software was used to integrate a final urban area map and inundation flood zones to determine the most exposed areas.

The methodology adopted in this study is given in Figure 3. Three main steps were followed: (1) the WMS model was used to extract the drainage networks and basin characteristics, followed by the HEC-HMS, which has a high capability of calculating hydrograph curves in multiple ways, according to ease of basin drainage, by either natural or artificial methods; (2) a hydraulic model (HEC-RAS) was used, which has a high capacity for determining the runoff limits, depths, and velocities of flooding; and (3) spatial modeling (GIS and RS) was used, with a high capacity for linking, identifying, and spatially analyzing areas at risk for flooding in the city, leading to sustainable urban development.

The rainfall depth had been estimated for different return periods (2, 5, 10, 20, 25, 50, or 100 years) by using a statistical analysis program [53], and various statistical distribution methods, such as normal, log-normal, log-Pearson type III, Pearson type III, Gumbel, and exponential, were applied to determine the best fit. To calculate the effective rainfall for each basin, it was necessary to use mathematical equations that represented rainfall loss or that linked runoff to total rainfall. Kirpich’s equation [54] was used to calculate the time of concentration (Table 1, Equation (1)), which is the time that passes between the rainfall and the highest level of floodwater going through the watershed area. Lag time, which is the time that passes between the occurrence of a unit of rainfall and a unit of runoff, was calculated according to Soil Conservation Service (SCS) guidance (Table 1, Equation (2)). To calculate the effective rainfall for each basin, it was necessary to use mathematical equations representing rainfall loss or linking runoff to total rainfall (Equations (3)–(5), Table 1). The depth of rain or direct flood in the basin was calculated to derive the total quantity of floodwater from the actual rain value using Equation (3) (Table 1). The amount of water in the area before the occurrence of flooding, such as filtration and suspended rain on plants, was estimated using Equation (4) (Table 1), and Equation (2) could be simplified as shown in Equation (5) (Table 1). The maximum effort for soil moisture (Sr; maximum retention in cm) was calculated from the curve number [55]. Peak discharge (m^3^/s) was calculated for each basin for different return periods using Equation (7) (Table 1), and the time to flood peak was calculated using Equation (8) (Table 1).

## 5. Results

### 5.1. Analysis of Rainfall Amounts in Water Basins of Tabuk City

**Figure 4:**
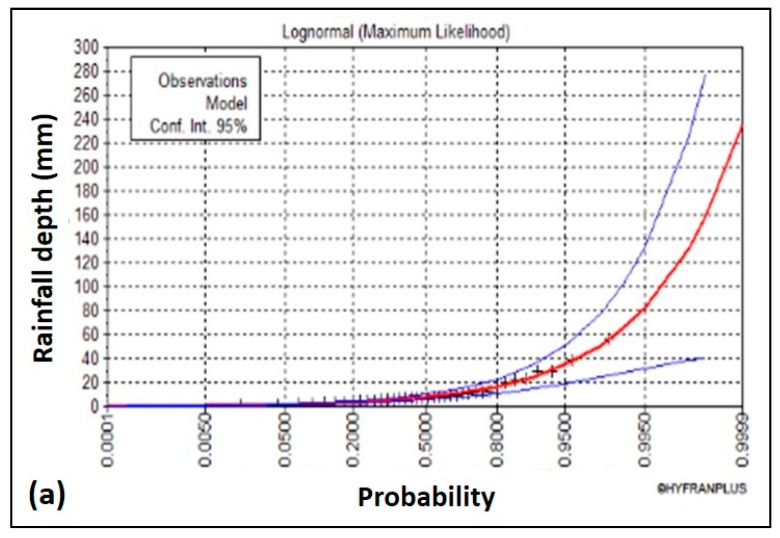

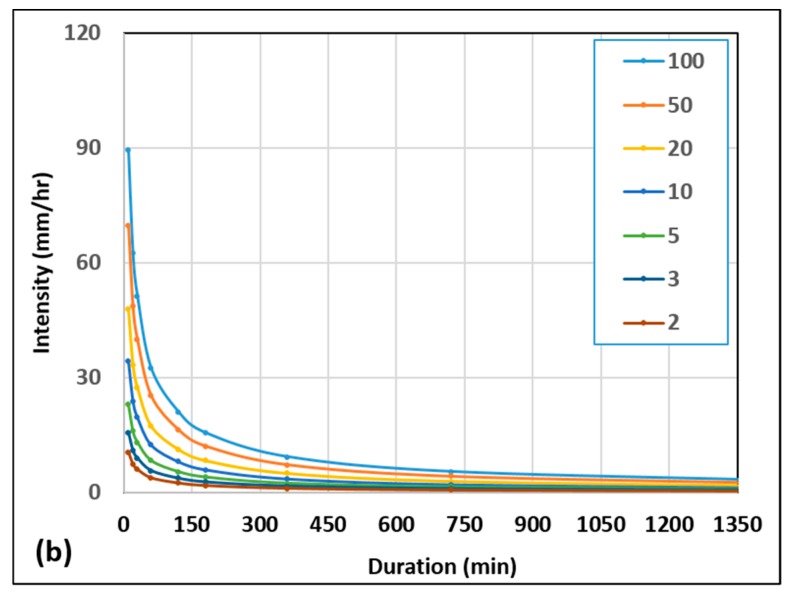
(**a**) Probability distribution curves at Tabuk Station (TB001) for maximum daily data using the log-normal method; (**b**) intensity–duration–frequency (IDF) curves of Tabuk City (TB001) intensity data during the period 1965 to 2004.

The records of the Ministry of Water and Electricity, General Presidency of Meteorology and Environmental Protection, and daily and hourly data that were available at Tabuk Meteorological Station (TB001) (Figure 1b) covered 1965 to 2004, about 38 years. The results indicated that the log-normal method was the best-fitting curve, as shown in Figure 4a. The rainfall intensity–duration–frequency (IDF) curves for different return periods were also established, as shown in Figure 4b. Table 2 shows the rainfall depths for different return periods.

### 5.2. Drainage Networks and Basins and Their Characteristics

**Figure 5:**
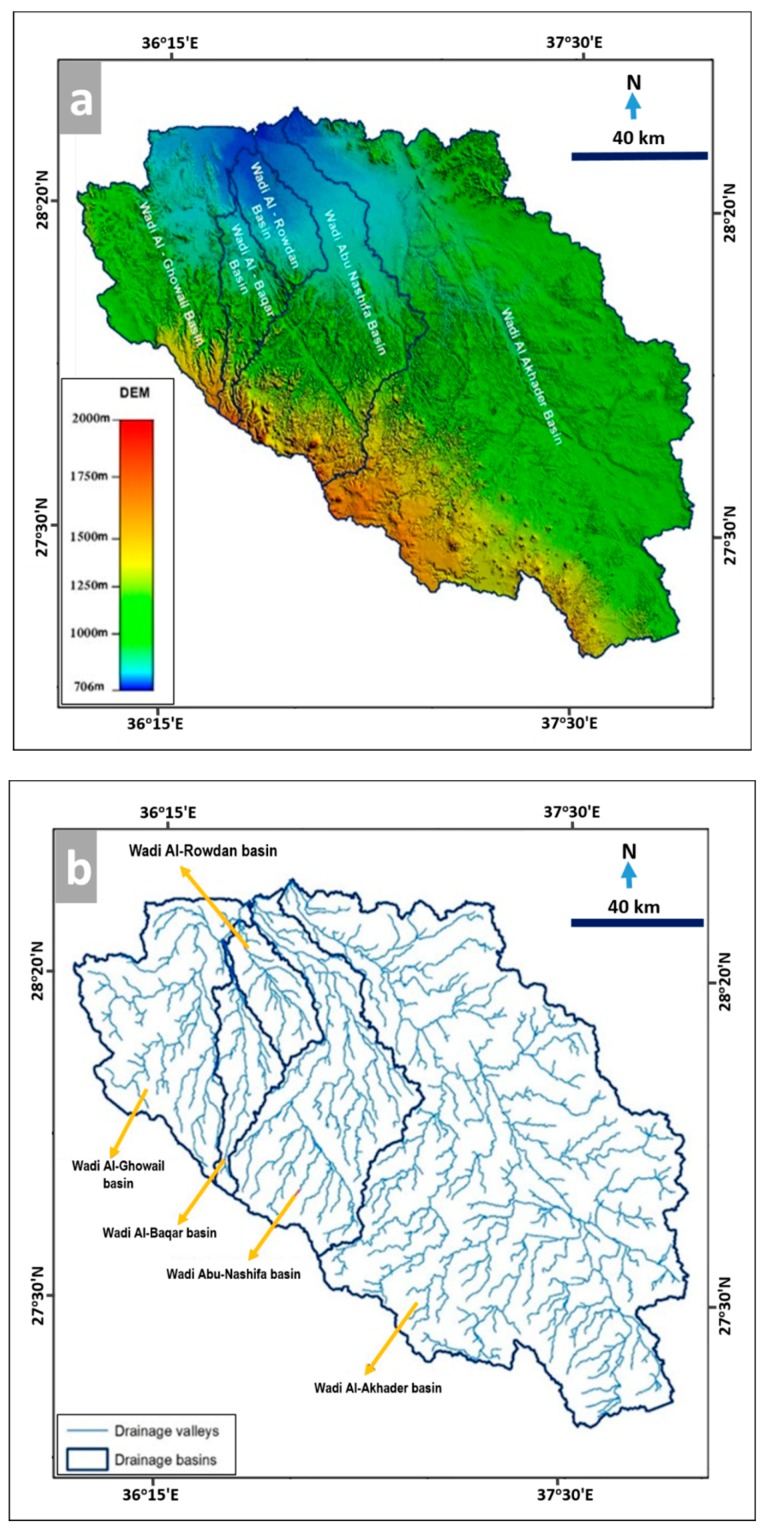

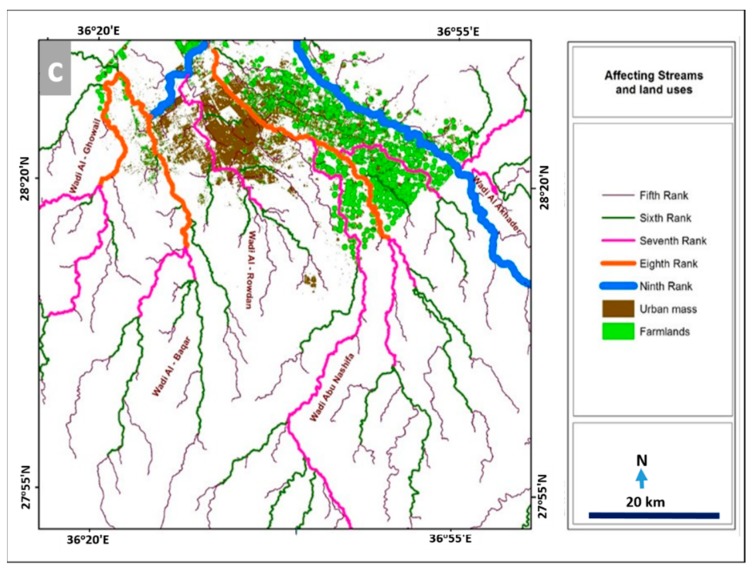
(**a**) The 5 m DEM of the study area, (**b**) extracted drainage networks and basins affecting Tabuk City, and (**c**) stream orders of wadis affecting the city in 2018.

To extract the drainage networks and basins that affect Tabuk City, several data sources were used, including a high-accuracy digital elevation model of 5 m resolution that was acquired from the Space Research Institute in King Abdul Aziz City for Science and Technology (Figure 5a). The WMS mathematical model was used to extract drainage networks and drainage basins in the study area (Figure 5b). Additionally, stream orders that affect Tabuk City are illustrated in Figure 5c. It was noticed that the city has expanded west and north toward Wadi Al-Baqar, Wadi Al-Rowdan, and Wadi Al-Ghowail, east toward Wadi Al-Akhader, and south toward Wadi Abu-Nashifa (Figure 5c). The main wadis affecting the study area have a number of tributaries that provide the majority of the flow toward them. The extracted streams and catchments were verified using topographic maps (1:50,000 scale) obtained from the Saudi Geological Survey. Twelve topographic sheets were scanned and rectified to create a topographic mosaic for the study area. Another validation was carried out for the wadis using recent satellite images (Landsat Land8/OLI 15 m resolution and Geo-Eye 1.84 m resolution). Additionally, various field investigations were completed to verify the accurate locations of the wadis, especially those running through the development boundaries of the city.

Different drainage basin characteristics were extracted and calculated, including morphometric parameters of the wadis, time of concentration, lag time, and curve number. These are shown in Table 3. 

**Figure 6:**
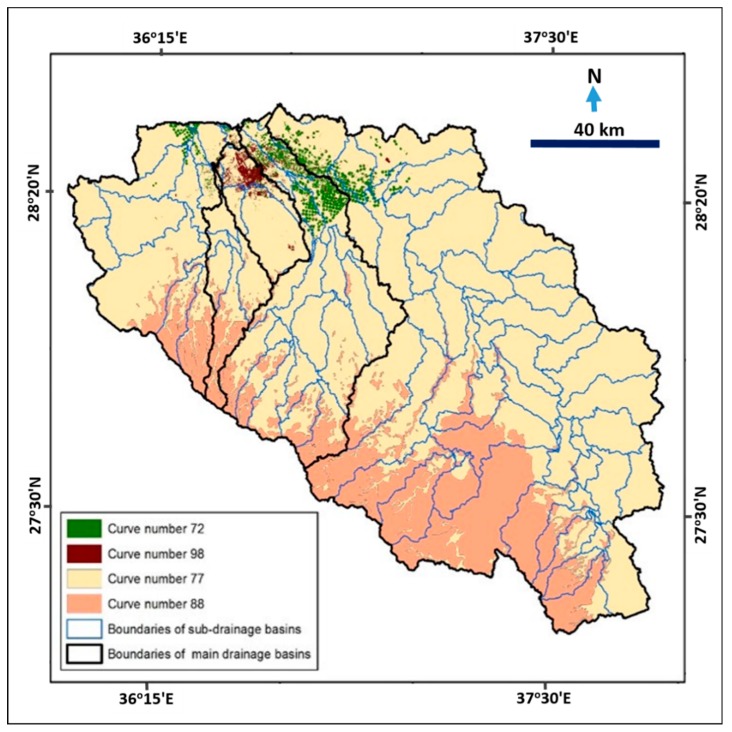
Combination map of soil type and land use units used to calculate curve number of drainage basins.

To calculate the effective rainfall for each basin, it was necessary to use mathematical equations that represent rainfall loss or link runoff to total rainfall. A land use map was obtained by applying supervised classification (maximum likelihood classification) on satellite images (Landsat Land8/OLI) in the Erdas Imagine program. A geological map was used to create the soil hydrologic property types. Both land use and soil maps were used to estimate the curve number value for each basin. The curve number (CN) method is commonly used to estimate quantities of water loss and the effective runoff value. Curve number ranges from zero to 100, reflecting the response of water to components of the land cover in drainage basins. Ponce et al. [56] indicated that the curve number value reflects the total area of impunctate surface, and as the value increases, the surface area decreases. Figure 6 illustrates a map of the curve number of drainage basins affecting Tabuk City.

### 5.3. Flood Analysis for Drainage Basins

Hydrologic calculations depending on stormwater design calculations were conducted using statistical analysis of rainfall at a rain station in the study area, and based on that, the maximum daily rainfall depth was determined for different return periods in each drainage basin. A mathematical hydrologic model (WMS, HEC-1) was used to transform rainfall data into flow at the basin outlet [57], and then the hydrologic flow values and volumes for each catchment were calculated (Table 4). To calculate drainage and runoff values, this depth is distributed by assuming the duration of the stormwater and determining the daily rainfall depth distribution curve during a stormwater design period to feed the model. Barkhordai et al. and Majidi et al. [58,59] applied the SCS unit hydrograph method to estimate the runoff from rainfall for the catchment area. The SCS unit hydrograph method is a powerful approach to determining flow hydrograph values based on a unit hydrograph method. It transforms rainfall values to runoff through a convolution procedure. Stormwater design distributions have been used, known worldwide as SCS, in which the duration of the stormwater is assumed to be 24 h. The distribution of SCS-TYPE II was chosen as appropriate for arid and semi-arid areas, where it assumes about 60% of the rainfall depth with daily rainfall in less than 2 h, and the rest is distributed throughout the day [55]. A crucial phenomenon of the model is that it detects variations in peak discharge and water volume from one sub-basin to another due to variations in the depth of soil moisture storage capacity across a catchment area and changes in morphometric characteristics and geological properties. Therefore, the catchment response to rainfall will change as these stores become saturated.

### 5.4. Inundation Areas Mapped Using HEC-RAS

**Figure 7:**
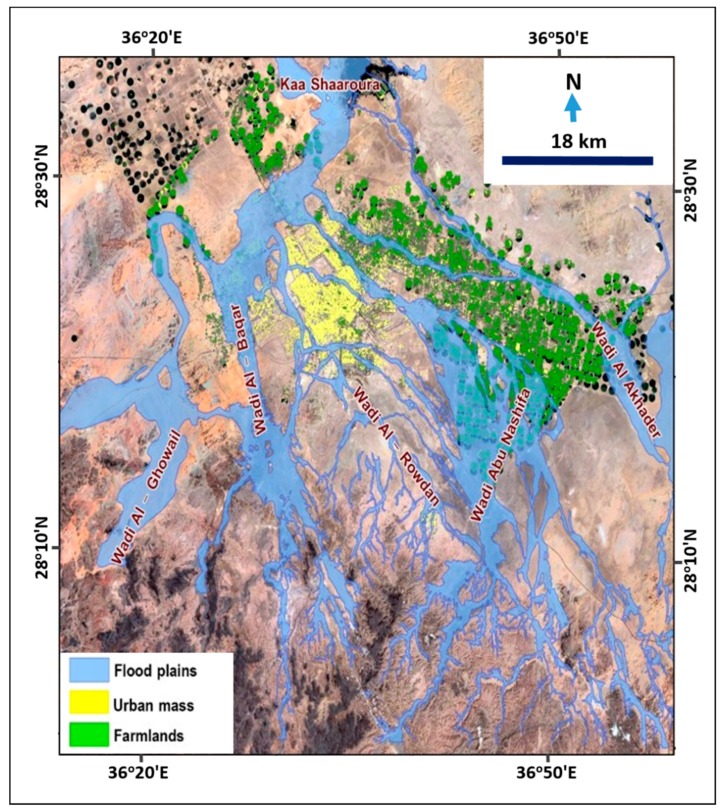
Inundation floodplain and its relationship to Tabuk urban areas in 2018.

A hydraulic model was built on the HEC-RAS program to identify the level of water along the floodplain of the wadis that have a significant impact on Tabuk City. A digital elevation model (5 m spatial resolution) was used to create different cross-sections along the wadi courses to help determine the width of runoff (extent of inundation areas), water depth, and water velocity. Figure 7 illustrates the flood limits (flood inundation areas) of the valleys affecting the city.

### 5.5. Land Use Changes (Urban and Agricultural Areas) 

In the current study, agricultural area changes were detected using the normalized difference vegetation index (NDVI) method. Supervised classification was done using ENVI 5.4 software, based on ground truth data and digital topographic maps of the study area. The resulting map was verified using visual interpretation in a GIS environment. 

**Figure 8:**
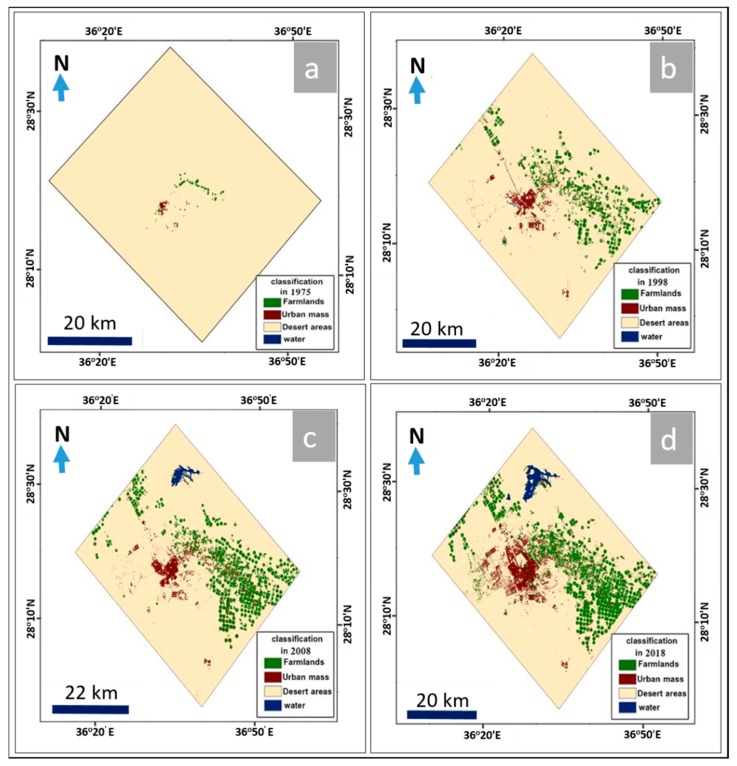
Urban and agricultural changes between 1975 and 2018 (**a**–**d**) in the Tabuk area, extracted from satellite images using Erdas Imagine and ArcGIS.

**Figure 9:**
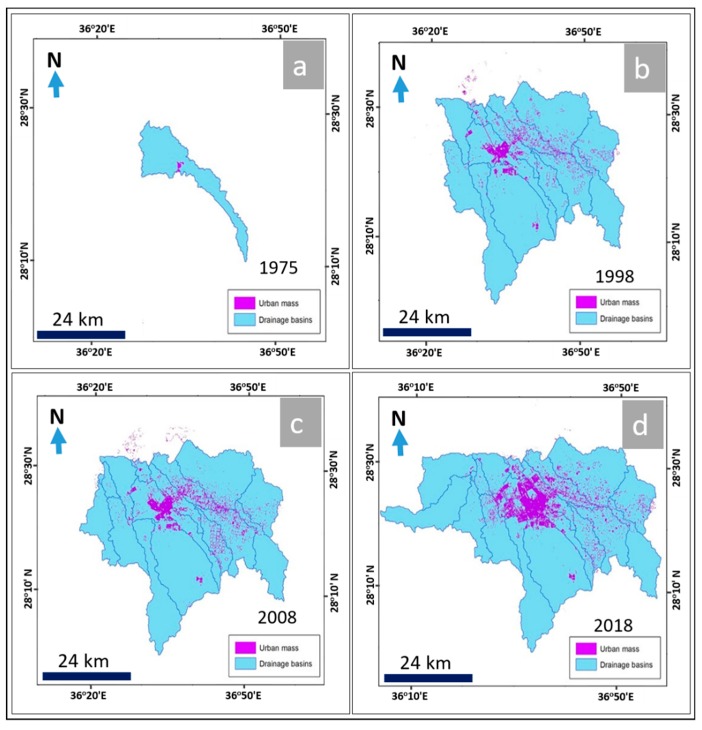
Relationship between urban expansion and drainage basins during the period 1975 to 2018 (**a**–**d**).

The results indicated that the classification of Landsat 8/OLI satellite images had two clear dimensions. The first was a quantitative calculation and analysis of values of urban growth, including urban area, average annual increase, and urban growth rates. The second was where spatial elements [43] of urban growth were analyzed by tracking the axes of urban growth. Figure 8 illustrates the changed features of land uses in both urban and agricultural areas of Tabuk City. The multitemporal data from the Landsat Thematic Mapper (TM, 30 m spatial resolution), Landsat Enhanced Thematic Mapper Plus (ETM+, 15 m spatial resolution), and Landsat OLI images (15 m spatial resolution) were processed using spatial analysis tools (resampling, georeferencing, classification, and post-classification overlay) to map the patterns and extent of urban and agricultural area changes and determine the magnitude of those changes at different time periods (1975, 1998, 2008, and 2018) (Figure 8). The classification results obtained from the RS images were compared and verified with the agricultural and urban distributions through the use of Geo-Eye images (1.84 m spatial resolution, acquired in June 2018). The results when using these sets of images indicated that the urban areas showed uncontrolled spreading, from covering an area of approximately 1.59 km^2^ in 1975 to an area of about 50.36 km^2^ in 1998, then an area of about 64.35 km^2^ in 2008, and finally, urban development covered an area of approximately 118.35 km^2^ in 2018 (Figure 9). Similarly, agricultural areas increased over time as well, from covering an area of about 5.8 km^2^ in 1975 to an area of approximately 136.8 km^2^ in 1998, then an area of about 221.9 km^2^ in 2008, and finally reaching an area of about 281.1 km^2^ in 2018 (Table 5 and Figure 8).

**Figure 10:**
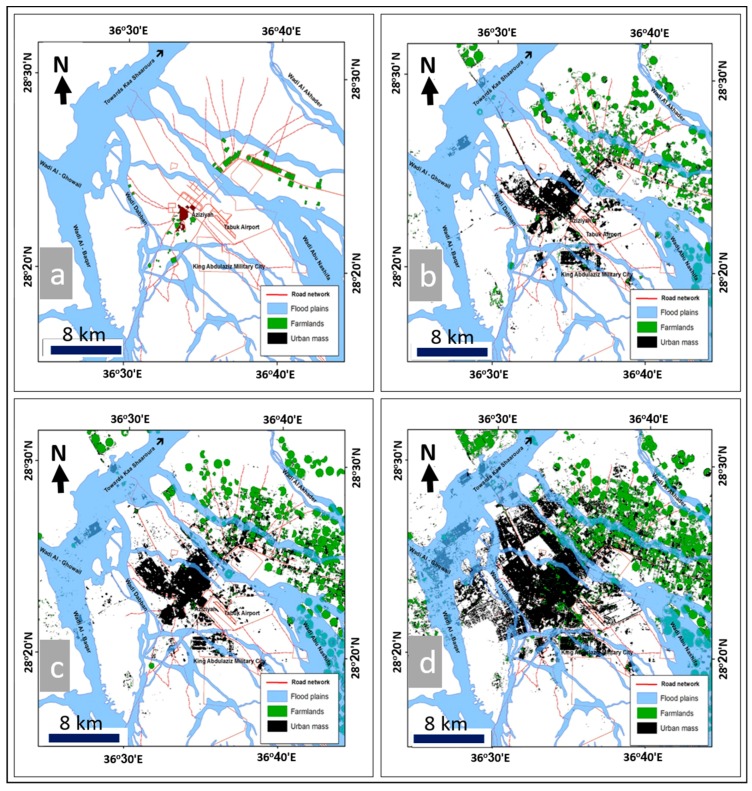
Urban areas of Tabuk City exposed to flooding hazards during the period 1975 to 2018 (**a**–**d**).

From that data, it can be seen that between 1975 and 1998, the annual increase in the area of sub-basins that contained urban distributions was approximately 83.86 km^2^ per year, with a variation rate of about 962.4%. The annual increase of the sub-basin area prone to flash flooding was approximately 12.81 km^2^, with a variation rate of about 1200%. Additionally, the annual increase in the distribution of urban development was about 2.12 km^2^ per year, with a variation rate of approximately 3061% (Table 6, Figure 9 and Figure 10).

During the period from 1998 to 2008, the annual increase in sub-basin area with urban distributions was approximately 15.55 km^2^ per year, with a variation rate of 7.3% per year. The annual increase in sub-basin areas prone to flash flooding was about 7.37 km^2^ per year, with a variation rate of approximately 23.1%, and the annual increase in urban mass distribution in these sub-basins was approximately 13.97 km^2^ per year, with a variation rate of about 27.7% (Table 6, Figure 9 and Figure 10).

Finally, between 2008 and 2018, the annual increase in sub-basin area that included urban development was approximately 36.22 km^2^ per year, with a variation rate of about 15.9%. The annual increase in sub-basin areas prone to flash flooding was about 4.91 km^2^ per year, with a variation rate of around 12.5%. Finally, the annual increase in urban distribution in these sub-basins was approximately 5.42 km^2^ per year, with a variation rate of about 84.3% (Table 6, Figure 9 and Figure 10). 

Field investigation and data extracted from the flood hazard maps indicated that most of the urban areas exposed to flooding hazards are concentrated in neighborhoods in the northwest part of the city and among new plans to the west, encompassing around 50% of the total urban areas. These portions of the city have a total population of 516,000 inhabitants, according to the General Authority for Statistics (2017). The affected neighborhoods include Al Tawain, Al Quads, Khrnata, Al Shifaa, Al Ain, Al Jamaa, and Al Boady. The eastern neighborhoods comprise about 30% of the urban areas exposed to flood hazards, representing approximately 40% of the city’s total population. They include the neighborhoods of Al Hamraa, Al Rabea, Al Rawaat, Al Sheikh, Al Khalidiya, Al Houdaiba, Al Ghawaysha, Al Saadah, and Al Salam. The remaining 20% of the affected urban area is located in the middle of the city and houses about 15% of the city’s population. This encompasses the neighborhoods of Al Morooj, Iskan of security forces, Al Maseef 1, Al Morooj 2, Al Rayan, Al Rabieh, and Al Maseef 3.

## 6. Discussion

### 6.1. Relationship between Urban Expansion, Basins, and Flood-Prone Areas

Several studies have been carried out to evaluate the impact of land use changes on runoff amounts [60,61,62,63,64]. Also, many authors have acknowledged the fact that increasing urban activities in flood plain areas will increase peak discharge, decrease the time to peak, and increase runoff volume [14,15,65,66,67]. A better understanding and evaluation of land use changes that have a direct impact on watershed hydrologic processes has become crucial for planning, management, and sustainable development of the watershed [68,69,70,71]. Those studies used land use changes to predict flood potential and mitigation of flood hazards. Recently, remote sensing and GIS have been used as powerful and effective tools to determine land use changes, map flood inundation, and assess flood risk using satellite images with the help of hydrologic and hydraulic models [72,73,74,75,76].

In the current study, ArcGIS 10.2 was used to extract the quantitative information based on the results of hydrologic (WMS) and hydraulic (HEC-RAS) modeling and the interpretation of RS images completed with Erdas Imagine with regard to the current sections of urban expansion and how they have been impacted by flash flood hazards at different times. Four stages were analyzed to understand the urban changes, the sub-basins that are impacted by urban distributions, and the sub-basins areas that are prone to flash flooding (Table 5 and Table 6, Figure 9 and Figure 10). Field observations and measurements have verified the changes in urban and agricultural areas between 1975 and 2018.

The data analysis showed that in the first stage, around the end of 1975, the urban distribution covered an area of about 1.59 km^2^, located in one sub-basin that covered an area of approximately 200.42 km^2^. The sub-basin contained an area prone to flooding of about 24.56 km^2^.

The second stage, around the end of 1998, showed that the urban mass was distributed over an area of approximately 50.37 km^2^ located in 13 sub-basins, which covered an area of about 2129.27 km^2^. The area prone to flooding in the sub-basin was approximately 319.28 km^2^.

In the third stage, around the end of 2008, the urban spread was distributed over an area of about 64.34 km^2^ located in 16 sub-basins, which had an area of approximately 2284.78 km^2^, and the area of sub-basins that was prone to flooding was about 392.96 km^2^.

Finally, in the fourth stage, around the end of 2018, the data showed that the urban spread was distributed over an area of approximately 118.58 km^2^ located in 18 sub-basins, with an area of about 2646.93 km^2^. The sub-basins had an area prone to flooding of approximately 442.08 km^2^.

### 6.2. Protection and Flood Hazard Prevention Plan for the City

The unplanned urban encroachment in the study area experienced severe flash floods in 2008, 2013, and 2016. These repeated events warn us of the need to develop a flood management system to protect the city from future flash floods by applying suitable and robust corrective measures. 

**Figure 11:**
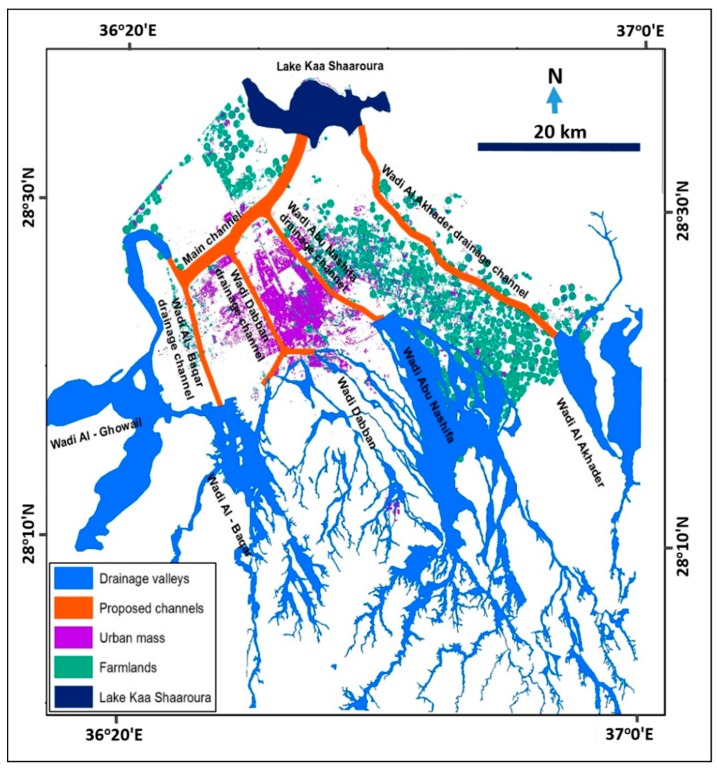
Map of proposed protective channels for prevention of flood hazards in Tabuk City.

This study proposes the implementation of six transformative open channels to contain and control the floodwaters coming from the wadi courses of Al-Akhader, Abu Nashifa, Dabban, Al-Baqar, and Al-Ghowail (Figure 11). The proposed channels will protect Tabuk City from future flood events. They should be designed to accommodate the maximum flow of a 100-year return period (Table 4). The final destination of the six channels will be the lake of Kaa Sharouraa, located to the north of the city.

## 7. Recommendations

The recommendations made based on this study prioritize the implementation of a flood hazard prevention and protection plan by constructing a group of conversion channels. Such construction should take place in phases, taking into account the urban areas that are exposed to flood hazards: the northwest neighborhoods and new western schemes (comprising about 50% of the total urban area), the eastern neighborhoods of the city (approximately 30% of the total urban area), and the city center areas (about 20% of the total urban area).

The results of this study highlight the need to cease urban expansion toward the northwest, in the direction occupied by Wadi Al-Ghwail and Wadi Al-Baqar, and to the east toward Wadi Al Khader. No authority is permitted to divide, plan, develop, or use any land within or outside the boundaries of urban development unless it receives approval for the land scheme from the Ministry of Municipal and Rural Affairs and the subsequent approval of hydrographic studies conducted to prevent the danger of flooding due to any such project.

Thus, this study suggests that it is important to apply the Council of Minister’s decision dated 21 May 2007, which sets out controls and procedures to be taken in response to flood damage, and adopt hydrologic and engineering designs necessary for flood drainage before adopting housing or agricultural plans. It is also recommended that no work permits be granted in the valleys unless the work is done in coordination with the relevant authorities.

Environmental sustainability is one of the requirements for balanced development, and this will be achieved only through the conservation and environmental rehabilitation of the valleys by utilizing water and resources to contribute to the protection of water, food, and housing security in order to establish sustainable urban development and mitigate negative environmental effects.

Given the results of this study showing the wadis included as drainage paths for floodwaters, environmental rehabilitation of the wadis could involve converting them into attractive recreational and tourist sites, with paths and open parks for a wide range of use by picnickers and lovers of nature.

Applying the hydrologic and hydraulic models of WMS, HEC-HMS, and HEC- RAS in flood risk prevention and urban environment management studies is crucial. Integrating these techniques with spatial modeling programs (GIS and RS) has proven to be highly effective for identifying which urban areas are exposed to flood hazards and contributing to urban environmental management and risk mitigation.

This study produced a spatial database during different stages that can be utilized to present the results to decision-makers to help them implement plans for preventing and controlling flood hazards and to help in the development and improvement of an efficient rainwater drainage infrastructure in conjunction with the KSA’s Future Vision 2030.

## 8. Conclusions

Tabuk City is one of the Saudi cities pursuing urban expansion that has encroached on the flow paths of wadis, a prominent phenomenon with the city’s expansion. The urban areas around Tabuk City grew from 1.59 km^2^ in 1975 to 118.58 km^2^ in 2018, an average increase of 2.72 km^2^ per year, and significant portions of these urban areas are exposed to flood hazards. The expansion to the west, north, east, and south has led to the city occupying significant portions of Wadi Al-Baqar, Wadi Al-Ghowail, Wadi Al-Akhader, and Wadi Debaan. The absence of hydrologic studies conducted for most urban plans, along with unimplemented rainwater drainage systems and flood projects that take into account actual dimensions and accurate pathways of the main wadis, increase the risk of flooding. This is leading to a rise in problems, such as difficulty with drainage of water in the wadis, water bonding, and inundation of some neighborhoods, causing loss of life and property. Evidence of improper urban expansion was noticed during recurrent dangerous flooding in the city in 2008, 2013, and 2016, which, in turn, led to this research. Finally, this research could help the decision makers of Tabuk City to understand the impact of flash floods on uncontrolled urban expansion. In addition, it provides an alternative solution that could be studied and implemented to protect the city from future floods. 

## Figures and Tables

**Table 1:** Equations used in the current study.

Equation	Formula	Description
(1)	$t_{c}=0.0195\left(\frac{L^{0.77}}{S^{0.385}}\right)$	t_c_ = time of concentration (min); L = maximum flow distance (m); S = maximum flow distance slope (%); T_LAG_ = lag time (hour); Sr = maximum effort for soil moisture (maximum retention) calculated from curve number (cm); Y = basin slope (%); P = rainfall for different return periods (cm); Ia = amount of water before occurrence of flood, such as filtration and suspended rain on plants; qp = peak discharge (m^3^/s); A = basin area (km^2^); T_p_ = time to peak (hour); Q = direct runoff (mm); Δt = duration of designed stormwater
(2)	$T_{LAG}=\frac{L^{0.8}{\left[Sr+1\right]}^{0.7}}{1900\sqrt{Y}}$	
(3)	$Q={\left(P-\mathrm{Ia}\right)}^{2}/\left(P-\mathrm{Ia}+\mathrm{Sr}\right)$	
(4)	Ia=0.2Sr	
(5)	$Q={\left(P-0.2Sr\right)}^{2}/\left(P+0.8S\right)$	
(6)	$Q={\left(P-0.2Sr\right)}^{2}/\left(P+0.8\mathrm{Sr}\right)$	
(7)	$\mathrm{qp}=\frac{0.208\mathrm{AQ}}{\mathrm{Tp}}$	
(8)	T_p_ = Δt/2 + T_LAG_	

**Table 2:** Rainfall depths of different return periods at Tabuk station (TB001).

Return period (year)	2	3	5	10	20	50	100
**Rainfall within 24 h (mm)**	7.74	11.5	16.8	25.1	35	51	65.5

**Table 3:** Main morphological characteristics of the drainage basins.

Basin Name	Basin Area (km^2^)	Maximum Flow Distance (km)	Basin Slope (m/m)	Lag Time (min)	Time of Concentration (min)
Wadi Dabban	603.8	43.9	0.03010	166.8	278.0
Wadi Al-Baqar	658.9	72.9	0.12780	141.2	235.3
Wadi Al-Ghowail	2343.5	81.5	0.07940	184.7	307.9
Wadi Abu Nashifa	2771.3	103.9	0.11490	193.2	321.9
Wadi Al-Akhader	10,345.7	188.2	0.06110	389.3	648.8

**Table 4:** Characteristics of floods in main drainage basins for different return periods.

Basin Name	Description	Return Periods (years)				
		5	10	20	50	100
**Wadi Dabban**	Peak discharge (m^3^/s)	1.69	15.42	46.07	118.80	201.26
	Flood volume × 1000 (m^3^)	113.80	1102.50	3277.40	8327.20	13,981.80
**Wadi Al-Baqar**	Peak discharge (m^3^/s)	3.32	24.59	72.64	187.23	317.33
	Flood volume × 1000 (m^3^)	171.00	1357.60	3860.50	9563.70	15,885.60
**Wadi Al-Ghowail**	Peak discharge (m^3^/s)	3.81	46.37	145.71	385.36	659.65
	Flood volume × 1000 (m^3^)	286.40	3700.40	11,626.80	30,450.40	51,781.00
**Wadi Abu Nashifa**	Peak discharge (m^3^/s)	4.52	54.98	172.77	456.94	782.25
	Flood volume × 1000 (m^3^)	338.90	4375.70	13,748.70	36,007.80	61,231.30
**Wadi Al-Akhader**	Peak discharge (m^3^/s)	17. 03	145.32	421.92	1058.90	1768.41
	Flood volume × 1000 (m^3^)	2354.50	20,252.80	58,670.80	146,911.70	245,146.60

**Table 5:** Urban and agriculture expansion in different time periods.

Factor	Year			
	1975	1998	2008	2018
**Number of sub-basins intersecting urban areas**	1	13	16	18
**Area of sub-basins including urban areas (km^2^)**	200.42	2129.27	2284.78	2646.93
**Area of flood-prone areas in sub-basins (km^2^)**	24.56	319.28	392.96	442.08
**Agricultural area distribution (km^2^) (Figure 5)**	5.80	136.80	221.90	281.13
**Area of urban distribution in sub-basins (km^2^) (Figure 6)**	1.59	50.37	64.35	118.58

**Table 6:** Urban and agriculture changes between 1997 and 2018.

Factor	Annual Increase between 1975 and 1998/Variation Rate (%)	Annual Increase between 1998 and 2008/Variation Rate (%)	Annual Increase between 2008 and 2018/Variation Rate (%)
**Number of sub-basins intersecting urban areas**	0.52/1200%	0.3/23.1%	0.2/12.5%
**Area of sub-basins including urban areas (km^2^)**	83.86/962.4%	15.55/7.3%	36.22/15.9%
**Area of flood-prone areas in sub-basins (km^2^)**	12.81/1200%	7.37/23.1%	4.91/12.5%
**Agricultural area distribution (km^2^) (Figure 5)**	5.70/2258.6%	8.51/62.2%	5.92/26.7%
**Area of urban distribution in sub-basins (km^2^) (Figure 6)**	2.12/3061%	1.39/27.7%	5.42/84.3%
